# Development of Spermatogenesis In Vitro in Three-Dimensional Culture from Spermatogonial Cells of Busulfan-Treated Immature Mice

**DOI:** 10.3390/ijms19123804

**Published:** 2018-11-29

**Authors:** Ali AbuMadighem, Ronnie Solomon, Alina Stepanovsky, Joseph Kapelushnik, QingHua Shi, Eckart Meese, Eitan Lunenfeld, Mahmoud Huleihel

**Affiliations:** 1 The Shraga Segal Department of Microbiology, Immunology, and Genetics, Beer-Sheva 84105, Israel; abumadig@post.bgu.ac.il (A.A.); ronnieso@post.bgu.ac.il (R.S.); alinaste@post.bgu.ac.il (A.S.); 2 The Center of Advanced Research and Education in Reproduction (CARER), Faculty of Health Sciences, Beer-Sheva 8410501, Israel; kapelush@bgu.ac.il (J.K.); lunenfld@bgu.ac.il (E.L.); 3 Faculty of Health Sciences, Ben-Gurion University of the Negev, Beer-Sheva 8410501, Israel; 4 Department of Pediatric Oncology and Department of Hematology, Soroka University Medical Center, Beer-Sheva 8410501, Israel; 5 Molecular and Cell Genetics Laboratory, The CAS Key Laboratory of Innate Immunity and Chronic Disease, Hefei National Laboratory for Physical Sciences at Microscale and School of Life Sciences, University of Science and Technology of China, Hefei 230000, China; qshi@ustc.edu.cn; 6 Institute of Human Genetics, Saarland University, Homburg/Saar, 66421 Homburg, Germany; eckart.meese@uks.eu; 7 Fertility and IVF Unit and Department of Obstetrics and Gynecology, Soroka University Medical Center, Beer-Sheva 8410501, Israel

**Keywords:** chemotherapy, male infertility, spermatogenesis, testis, three-dimensional culture, spermatogonial cells

## Abstract

Aggressive chemotherapy may lead to permanent male infertility. Prepubertal males do not generate sperm, but their testes do contain spermatogonial cells (SPGCs) that could be used for fertility preservation. In the present study, we examined the effect of busulfan (BU) on the SPGCs of immature mice, and the possible induction of the survivor SPGCs to develop spermatogenesis in 3D in-vitro culture. Immature mice were injected with BU, and after 0.5–12 weeks, their testes were weighed and evaluated histologically compared to the control mice. The spermatogonial cells [Sal-like protein 4 (SALL4) and VASA (a member of the DEAD box protein family) in the testicular tissue were counted/seminiferous tubule (ST). The cells from the STs were enzymatically isolated and cultured in vitro. Our results showed a significant decrease in the testicular weight of the BU-treated mice compared to the control. This was in parallel to a significant increase in the number of severely damaged STs, and a decrease in the number of SALL4 and VASA/STs compared to the control. The cultures of the isolated cells from the STs of the BU-treated mice showed a development of colonies and meiotic and post-meiotic cells after four weeks of culture. The addition of homogenates from adult GFP mice to those cultures induced the development of sperm-like cells after four weeks of culture. This is the first study demonstrating the presence of biologically active spermatogonial cells in the testicular tissue of BU-treated immature mice, and their capacity to develop sperm-like cells in vitro.

## 1. Introduction

Spermatogenesis is the process of proliferation and differentiation of spermatogonial stem cells (SSCs) to generate sperm. SSCs are located at the periphery of the seminiferous tubules close to the basement membrane [1,2,3,4,5,6,7,8]. The relative number of these cells compared to the testicular germ cells is very low—only around 0.03% [9,10]. While the rate of division of SSCs is very low, the division of their progeny is very high [3,6,8]. Therefore, the progeny is more affected by chemotherapy/ radiotherapy treatments, and anti-cancer or other disease treatments may lead to male infertility. Thanks to efficient treatment protocols, around 80% of chemotherapy/radiotherapy-treated patients now survive the disease [11,12,13,14,15]. 

Busulfan (BU) is an alkylating agent (leading to the formation of DNA–DNA and DNA–protein cross-links and single strand breaks) that is used in chemotherapy for the treatment of various cancers, and before bone marrow transplantation [16,17,18]. However, it causes gonadal dysfunction, kills mainly spermatogonial cells, and even may lead to azoospermia [19]. In addition, the functionality of testicular somatic cells may be affected by chemotherapy/radiotherapy treatments [20,21,22,23,24]; this may affect the SSCs’ microenvironment and niches, and, thus, is involved in male infertility. Indeed, BU treatment is used to deplete the spermatogonial cells in adult mice, which will be used in germ cell transplantation technology [19,25,26,27]. It was shown that the restoration of spermatogenesis in BU-treated adult mice was dose-dependent when the aggressive effect was in a dose of 45 mg/kg [24]. In addition, it was shown that the GDNF (glial cell line derived nerve growth factor) levels increased under these conditions [24]. However, the effect of BU on the development of spermatogenesis in immature mice has not been examined. 

As prepubertal males do not produce spermatozoa, the cryopreservation of sperm for fertility is not an option. However, their testes do contain SSCs, which could be used for future fertility preservation before aggressive anti-cancer treatments. While germ cell and testicular tissue transplantation has been shown to generate fertile sperm in rodents and other animal species, this has not yet been shown in humans [28,29,30,31,32,33]. In addition, Sato et al. showed that the testicular organ culture produced fertile sperm in vitro [34]. Recently, our group showed the induction of mouse spermatogonial cells from immature mice to proliferation and differentiation to meiotic and postmeiotic stages, including the generation of sperm-like cells in three-dimensional (3D) in-vitro culture systems, such as in soft-agar culture systems (SACS) [35,36] and methylcellulose culture systems (MCS) [37]. Using a 3D in-vitro culture system (MCS), we also were able to induce the proliferation and differentiation of spermatogonial cells isolated from prepubertal monkeys to meiotic and postmeiotic cells, including the development of round-like spermatid cells [5]. Recently, we were able to induce human spermatogonial cells from prepubertal cancer patients so as to develop meiotic and postmeiotic cells that included the generation of sperm-like cells in MCS [38]. 

Our hypothesis is that the testicular cells from BU-treated immature mice contain biologically active spermatogonial cells, and these cells could be induced using a 3D in-vitro culture system (MCS) to proliferate and differentiate to the meiotic and postmeiotic stages that include sperm-like cells. Furthermore, these cells could be used in the future to develop embryos using intracytoplasmic sperm injection (ICSI) technology.

## 2. Results

### 2.1. Effect of BU on the Testicular Parameters of Immature Mice

**Figure 1:**
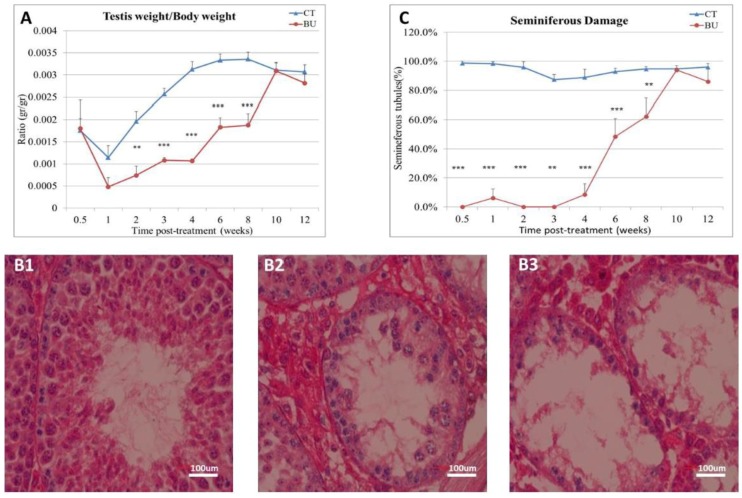
Effect of busulfan (BU) on testicular and body weight and on testicular histology: BU was intraperitoneally injected (i.p; 45 mg/kg in 100 µL). After 0.5–12 weeks, mice were weighed, and the testes were removed and weighed. Part of the testes were fixed in Bouin’s solution (for histological evaluation), while the other part was frozen at −70 °C for RNA extraction and/or for the preparation of total protein. The ratio of the testes weight/body weight after 0.5–12 weeks of BU injection compared to the control (CT) (DMSO) is presented (**A**). The histology of the seminiferous tubules after BU treatment was normal (**B1**), showed moderate damage (**B2**), or showed severe damage (**B3**). A summary of the seminiferous tubule damage after 0.5–12 weeks of BU treatment compared to the CT is presented (**C**). ×40 light microscope magnification (100 µm scale). ** *p* < 0.01 and *** *p* < 0.001.

Our results show that the treatment of immature mice with BU significantly decreased the testicular weight (as presented by the ratio of the testicular weight/testicular body) immediately, one week after BU injection, until eight weeks, as compared with the control (Figure 1A). After two weeks, a gradual increase in the testicular weight was observed equal to the control after 10 weeks (Figure 1A). In parallel, the BU injection led to damage in the histology of the seminiferous tubules (STs) of the testis (presented histology of the seminiferous tubules after six weeks of BU treatment), as follows: normal (Figure 1B1), moderate damage (Figure 1B2), and severe damage (Figure 1B3). The damage effect of the BU on the STs was significantly higher (the percentage of damaged STs) in parallel to the reduction in the testicular weight, while in the beginning, recovery of the STs was observed after six weeks to be similar to the control after 10 weeks (Figure 1C).

### 2.2. Effect of BU on VASA and SALL4 Spermatogonial Cells in Testicular Tissue of Immature Mice

**Figure 2:**
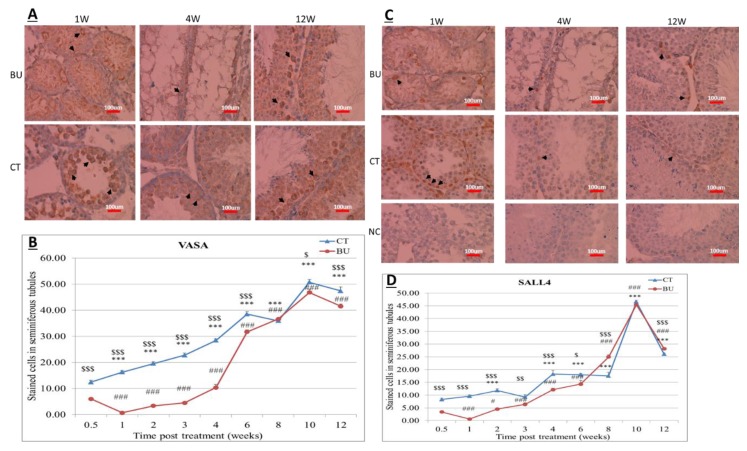
Effect of busulfan (BU) on VASA- and SALL4-positve cells in testicular tissue: BU was i.p injected, as described in Figure 1. Testicular tissue from different time points (1 week, 4 weeks, and 12 weeks) after the BU or control (CT) injection were examined for VASA- and SALL4-positive cells (**A** and **C**, respectively) using immunohistochemical staining. Negative control (NC) of the tissue is presented. Summary of the VASA-positive cell staining/tubule or SALL4-positive cell staining/tubule at different time points (0.5–12 weeks) after the BU or CT treatments is presented (**B** and **D**, respectively). × 40 light microscope magnification (100 µm scale). $ indicates comparison between control and treatment. * indicates comparison between weeks of control and first week of control. #—indicates comparison between weeks of BU-treatment and first week of BU-treatment. $$$, ***, ### *p* < 0.001, $$ *p* < 0.01, # and $ *p* < 0.05. Black arrows indicate the stained cells.

The testicular tissue from the BU-treated and control mice were prepared for VASA and SALL4 by immunohistochemical staining. Here, we present the results of staining from one and four weeks (with a severe effect of BU on the histology of the STs), and 12 weeks (with the recovery of the STs) after BU treatment (Figure 2A,C, respectively). Our results show a significant reduction in the stained cells of VASA and SALL4/seminiferous tubules 0.5–6 weeks after BU injection, as compared with the control (Figure 2B,D, respectively). A gradual increase in the number of VASA and SALL4 stained cells per seminiferous tubule was detected 2–12 weeks after BU injection, when they became similar to the control after eight weeks for VASA and SALL4 (Figure 2B,D, respectively).

In order to examine the effect of the BU treatment of immature mice on the capacity of their spermatogonial cells to develop spermatogenesis in vitro, we used immature mice after 10 days of BU treatment, the time point when, according to our results, there is a severe effect of BU (Figure 1 and Figure 2).

### 2.3. Effect of BU on Testicular Cell Count and Proliferation from Immature Mice 10 Days After Injection

**Figure 3:**
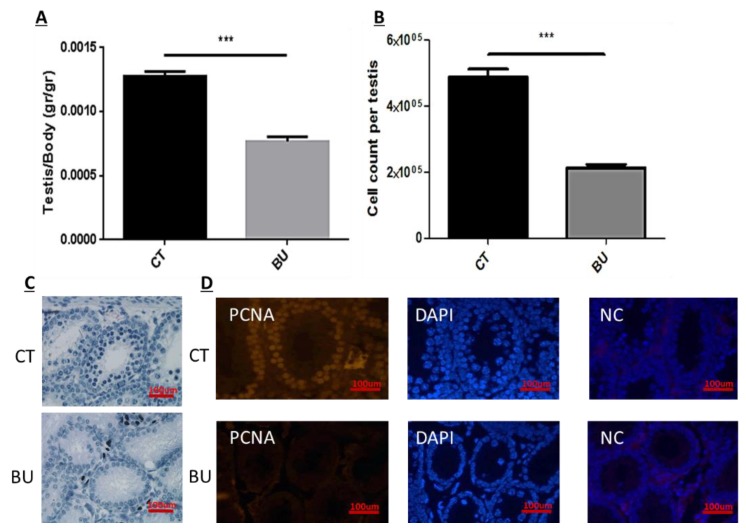
Effect of 10-day post busulfan (BU) treatment on testicular body weight, cell count, and proliferation: BU or dimethyl sulfoxide (DMSO) (control, CT) were i.p injected, as described in Figure 1. Ten days after the injection, the testes were weighed (**A**), the total cells in the seminiferous tubules were counted (**B**), the histology of testicular tissue was examined using hematoxylin and eosin (H&E) staining (**C**), and cell proliferation in testicular tissues was evaluated using proliferating cell nuclear antigen (PCNA) staining (**D**). Negative control (NC) of the tissue is presented. ×40 light microscope magnification (100 µm scale). *** *p* < 0.001.

Our results show that BU significantly decreased the testicular weight (presented as a ratio of testicular weight to body weight (*p* < 0.001) (Figure 3A) and testicular cell count compared with the control (CT) (*p* < 0.001) (Figure 3B). In addition, it damaged the seminiferous tubules compared with the control (Figure 3C), and significantly decreased the seminiferous tubule cell proliferation compared with the control (PCNA staining as an indicator of cell proliferation) (Figure 3D). 

### 2.4. Effect of BU on Subpopulations of Spermatogenic Cells from Immature Mice 10 Days after Injection

**Figure 4:**
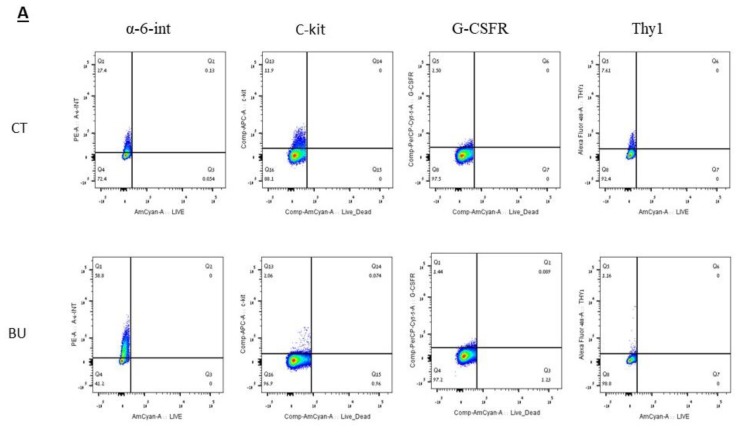

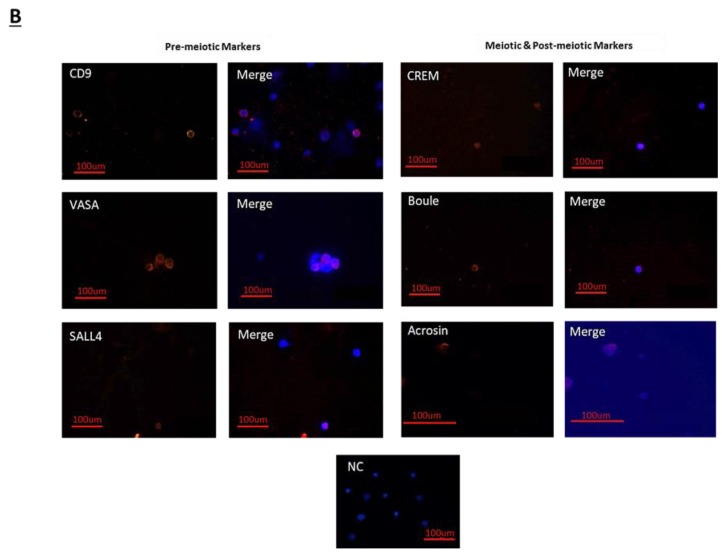

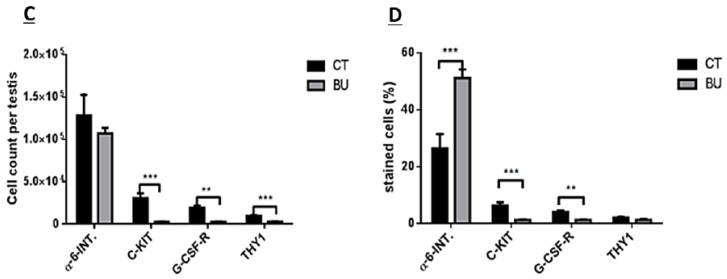
Effect of 10-day post busulfan (BU) treatment on spermatogonial cell counts and percentages, as examined by fluorescence-activated cell sorter (FACS) analysis, as follows: BU or DMASO (control, CT) were i.p injected, as described in Figure 1. Ten days after the injection, the testes were removed, seminiferous tubules were separated, and the cells were enzymatically isolated from the seminiferous tubules. Spermatogonial cells with the membrane markers: alpha-6-INTEGRIN (alpha-6-INT), c-KIT, G-CSF-R, and THY1 were identified by FACS using specific antibodies for each marker (**A**). Spermatogenic cells were identified by immunofluorescence staining for specific markers [premeiotic (CD9, VASA, and SALL4), meiotic (CREM, BOULE, and ACROSIN), and postmeiotic (ACROSIN)] (**B**). The above identified spermatogonial cells were counted and calculated per testicle (**C**), and their percentage was evaluated (**D**). ** *p* < 0.01 and *** *p* < 0.001.

**Figure 5:**
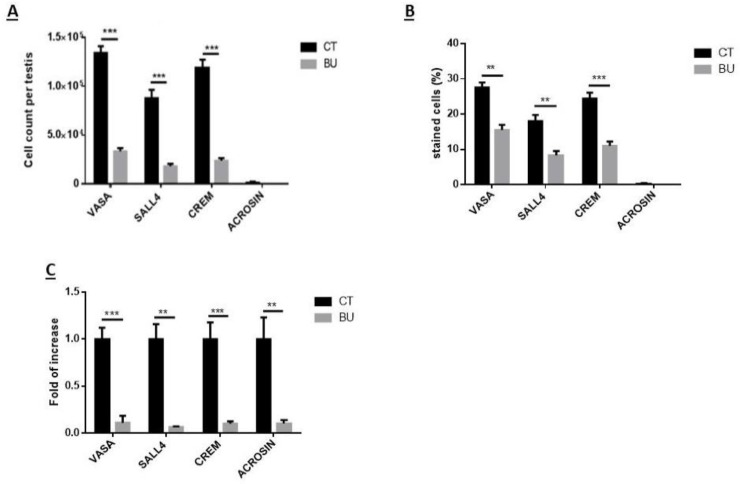
Effect of 10-day post busulfan (BU) treatment on spermatogenic cell counts, percentages and expression: BU or DMASO (control, CT) were i.p injected, as described in Figure 1. Ten days after the injection, the testes were removed, seminiferous tubules were separated, and cells were enzymatically isolated from the seminiferous tubules. The premeiotic (CD9, VASA, and SALL4), meiotic (CREM, BOULE, and ACROSIN), and postmeiotic (ACROSIN) cells were identified by immunofluorescence staining using the antibodies specific for each marker (Figure 4B). The above identified spermatogenic cells were counted and calculated per testicle (**A**), and their percentage was evaluated (**B**). Their expression was analyzed by qPCR analysis (fold of increase compared to control) (**C**). ×40 light microscope magnification (100 µm scale). ** *p* < 0.01 and *** *p* < 0.001.

We examined the effect of BU on subpopulations of spermatogonial cells according to cell membrane markers (by fluorescence-activated cell sorter (FACS) analysis; Figure 4A) and cytoplasmic/nuclear markers (by immunofluorescence staining; Figure 4B). Our results show that the injection of BU into immature mice did not affect the number of alpha-6-integrin positive cells, but significantly increased their percentage compared to the control (*p* < 0.001), as examined by FACS analysis (Figure 4C,D, respectively). However, the injection of BU into immature mice significantly decreased the number and percentage of C-KIT- (*p* < 0.001), G-CSFR- (*p* < 0.01), and THY-1-positive cells (*p* < 0.001), as examined by FACS analysis (Figure 4C,D, respectively). Using the immunofluorescence staining (Figure 4B) and qPCR analyses (Figure 5C), we could show that injection of BU to immature mice significantly decreased the cell number (Figure 5A), the percentages of the stained cells (Figure 5B), and the expression levels of the VASA and SALL4 cells (premeiotic cells), CREM-1 cells (meiotic cells), and ACROSIN (meiotic and postmeiotic cells) compared with the control (Figure 5C).

### 2.5. Effect of FSH, TNF, and Testicular Homogenates on the Capacity of Spermatogonial Cells from BU-Treated Immature Mice to Develop Spermatogenesis In Vitro

**Figure 6:**
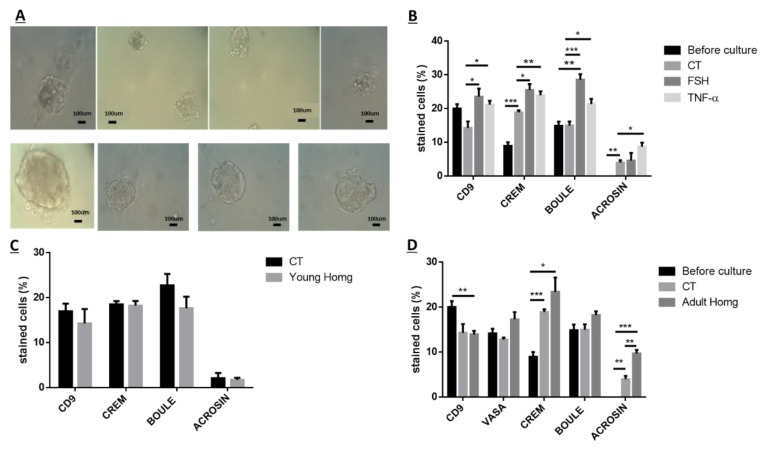

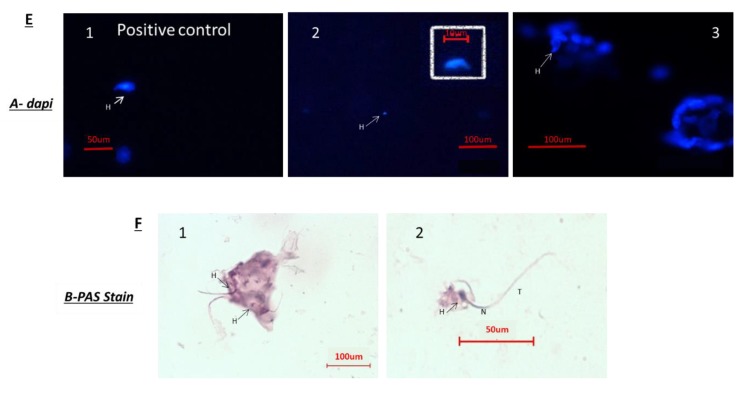
Development of spermatogenesis in vitro from BU-treated immature mice: Ten days after BU injection (see Figure 1), the testes were removed, seminiferous tubules were separated, and cells were enzymatically isolated and cultured in a methylcellulose culture system (MCS). The MCS was composed of 42% methylcellulose, KSR (10%), StemPro, and growth factors (GDNF (glial cell line derived nerve growth factor), LIF (leukemia inhibitory factor), FGF (fibroblast growth factor), and EGF (epidermal growth factor)) (see Methodology). In some wells, we also added TNF-α (20 pg/mL), FSH (7.5 IU/mL), or testicular homogenates from immature mice (60 µg/mL) or testicular homogenates from GFP-adult mice (60 µg/mL). Every 10–14 days, we added new media containing the same composition of factors that was added in the beginning of the culture. After 4–6 weeks, the developed colonies and cells (**A**) were collected, and the cells were fixed using cold methanol and were stained using immunofluorescence staining, using specific antibodies for markers of the premeiotic (CD9 and VASA), meiotic (CREM, BOULE, and ACROSIN), and postmeiotic (ACROSIN) cells. The effect of FSH and TNF-alpha (**B**) testicular homogenates from immature (**C**) or adult (**D**) mice on the development of spermatogenic cells in vitro compared to before the culture or the control (CT; after culture in the presence of growth factors) were examined. In addition, the development of the sperm-like cells in the cultures was examined according to the DAPI staining of sperm head morphology [**E**; E1—positive control of a sperm head (H); E2—a single sperm head developed in the culture; E3—a group of sperm heads developed in the culture] or periodic acid–Schiff (PAS) staining [**F**; F1—a group of sperm-like cells with heads (H) and tails; F2—a few sperm-like cells with complete morphology of head (H), neck (N), and tails (T)]. ×40 light microscope magnification (100 µm scale). * *p* < 0.05, ** *p* < 0.01 and *** *p* < 0.001.

The isolated spermatogonial cells from the seminiferous tubules of mice 10 days after BU treatment were cultured in vitro in methylcellulose (a 3D culture system) containing StemPro, KSR (knock-out serum replacement), LIF (leukemia inhibitory factor), GDNF, and FGF (fibroblast growth factor) without (control; CT) or with addition of follicle stimulating hormone (FSH) (7.5 IU/mL), TNF (20 pg/mL), or homogenates (60 µg/mL) from two-week-old GFP mice or six-week-old GFP mice for four weeks (see Materials and Methods section). Our results show that spermatogonial cells from BU-treated mice could proliferate (formation of clusters/colonies) in vitro (Figure 6A) and differentiate to meiotic and postmeiotic stages (generate CREM-1 and ACROSIN cells) under control conditions (CT), compared with before the culture (Figure 6B,D). The addition of FSH to the cultures showed a significant increase in the percentages of the CD9, CREM-1, and BOULE cells compared with the control (CT) (*p* < 0.0.5, *p* < 0.05, and *p* < 0.001, respectively), but did not affect the ACROSINE cell development (Figure 6B). The addition of TNF-α to the cultures significantly increased the percentages of the CD9, CREM-1, BOULE, and ACROSIN cells in the culture, compared with the control (CT) (*p* < 0.0.5, *p* < 0.01, *p* < 0.05, and *p* < 0.05, respectively) (Figure 6B). In addition, the addition of homogenates from two-week-old (young homogenates) mice did not significantly affect the percentages of the developed spermatogenic cells in the culture compared to the control (CT) (Figure 6C). However, the addition of homogenates from six-week-old (adult homogenates) mice to the cultures did not affect the percentages of the CD9, VASA, BOULE, and CREM cells, but significantly increased the percentages of the ACROSINE cells in the culture compared with the control (CT) (*p* < 0.001) (Figure 6D). In addition, the homogenates from adult mice induced the development of spermatogonial cells from BU-treated mice to generate sperm-like cells with complete morphology (head, neck, and tail) in vitro, as examined by immunofluorescence staining (Figure 6E) and PAS (Figure 6F). Some sperm samples were found to be individual (Figure 6E2) or in groups in the culture (Figure 6E3,F). 

## 3. Discussion

Our results show, for the first time, the presence of biologically active spermatogonial cells in the testes of immature mice treated with busulfan. These cells were able to proliferate and differentiate to meiotic and postmeiotic cells, and to develop sperm-like cells in vitro, using methylcellulose as a 3D in-vitro culture system.

The treatment of immature mice with BU significantly reduced their testicular weight for a period of eight weeks after the BU injection. This was in parallel with an increase in the percentage of the damaged seminiferous tubules. In addition, a significant reduction in the number of SALL4 and VASA cells/seminiferous tubules was observed during this period. The restoration of the testicular tissue weight began after two weeks, and was retained similar to that of the control after 10 weeks. This was in parallel to an increase in the number of SALL4 and VASA cells/seminiferous tubules and other developing spermatogenic cells (see histological sections). An increase in the number of normal seminiferous tubules was detected four weeks after BU injection. It is possible that the significant reduction in testicular weight could be related to the severe damage of the seminiferous tubules, and that restoration of these tubules increased the testicular weight. Thus, it is possible that even though the BU treatment of the immature mice led to severe damage of most of the seminiferous tubules and a reduction in their spermatogonial cell counts/seminiferous tubules, the survivor spermatogonial cells and somatic cells were still biologically active and capable of restoring the germinal epithelium of the seminiferous tubules. These results may suggest that, under some chemotherapy treatment conditions, when biologically active spermatogonial cells and somatic cells remain, it is possible that spermatogenesis may be restored. Our results show that the BU treatment of immature mice significantly decreased the different subpopulations of spermatogonial cells (α-6-INTEGRIN, c-KIT, G-CSF-R, THY-1, CD9, VASA, and SALL4 positive cells) 10 days after the injection. In addition, the cells that stained/expressed CREM-1 and BOULE (meiotic cells) and expressed protamine (RNA expression) were examined in the seminiferous tubules of the BU-treated immature mice, and were significantly lower compared to the control. However, the acrosin stained cells were not present, but their expression was significantly lower following BU treatment. Therefore, it is possible to suggest that after 10 days of BU treatment, some spermatocytes are still present and/or the spermatogonia has already started to differentiate in the seminiferous tubules to generate spermatocytes. It was demonstrated that spermatocytes express protamine RNA, but the process of its translation to protein is under suppression. Therefore, we suggest that, in our system, the spermatocytes are the source of the protamine RNA expression and not the elongated spermatids.

Thus, the effect of the BU treatment may lead to an imbalance in the factors produced by testicular somatic cells that are involved in the regulation of spermatogenesis, which leads to changes in the SSCs microenvironment and, as a result, may lead to impairment in the development process of spermatogenesis.

Because after BU treatment of immature mice, some biologically active spermatogonial cells survive the treatment, we used these cells (after 10 days of BU treatment) for the possible development of spermatogenesis in vitro in 3D in-vitro culture conditions. Our results show that these spermatogonial cells were able to proliferate (form colonies) after four weeks of culture in the presence of different growth factors (LIF, GDNF, FGF, and EGF) and denovo Sertoli cells. The addition of these factors that present in the testicles under physiological conditions, such as FSH or TNF-α, to MCS, significantly increased the proliferation of the spermatogonial cells and their differentiation to meiotic and postmeiotic cells, compared with the control (CT; without those factors). However, we could not identify the development of sperm-like cells under these conditions. As testicular tissue from adults contains various factors (endocrine and paracrine/autocrine factors) that together induce the development of complete spermatogenesis in vivo, we decided to add homogenates from sexually immature and adult mice (the composition of factors is different, and the stages of spermatogenesis are also different at these ages) to our in-vitro MCS. Our results show that the addition of testicular homogenates from adult mice significantly increased the capacity of spermatogonial cells from BU-treated immature mice (after 10 days of BU injection) to develop meiotic and postmeiotic cells compared to the control (CT; without addition of homogenates) in MCS. Under these conditions, we also were able to identify the development of sperm-like cells (appearance of sperm-like heads, as identified by DAPI, using IF staining) and sperm-like cells with complete morphology (head, neck, tail), as examined by PAS staining. Some of these cells were identified as individuals, while others were identified in clusters. These results may suggest the presence of biologically active spermatogonial cells in the testicular tissue of immature mice treated with BU. These spermatogonial cells were able to develop complete spermatogenesis, including the generation of sperm-like cells in MCS-contained testicular homogenates from adult mice. These results may suggest that these homogenates contain a combination of growth/differentiation and possibly endocrine factors, that together (in the suitable balance and ratio) were able to provide conditions in vitro similar to in-vivo conditions and, thus, to induce the development of complete spermatogenesis in vitro. The testicular homogenates were prepared from GFP mice, were frozen and thawed several times, and filtered before addition to the culture. This was performed to exclude the possibility that the identified sperm developed in MCS may be from the homogenates and not from the developed spermatogonial cells (the developed sperm was colorless, rather than green). On the other hand, the addition of homogenates from immature mice to spermatogonial cells from BU-treated mice did not affect the number of premeiotic, meiotic, and postmeiotic cells developed in MCS compared with the control (CT). These homogenates did not contain the factors present in the testes of adult mice and, therefore, could not induce the development of spermatogenesis similar to in-vivo conditions.

Thus, this is the first study that shows the presence of bioactive spermatogonial cells in the BU-treated immature mice with the capacity to develop complete spermatogenesis, including the generation of sperm-like cells under in-vitro culture conditions. These results are in harmony with our recent study that showed the presence of biologically active spermatogonial cells in the testes of chemotherapy-treated prepubertal boys with cancer. These cells were developed to meiotic and postmeiotic cells, including the generation of sperm-like cells in MCS [38]. Using 3D culture systems (MCS and/or SACS), we were able to induce the development of spermatogonial cells from normal immature mice (seven days old) to meiotic and postmeiotic cells, including the generation of sperm-like cells [35,36,37]. In addition, we were able to induce the development of spermatogonial cells from prepubertal monkeys to meiotic and postmeiotic cells in MCS [5]. In conclusion, our results suggest that MCS is a possible 3D system to induce the development of spermatogonial cells to meiotic and postmeiotic stages, including the generation of sperm-like cells. This system still needs optimization to increase the number of the developed cells (including sperm-like cells) and its efficiency. In addition, there is a need to evaluate the functionality of the developed sperm-like cells and their DNA quality, and its epigenetics. Therefore, in our institution, we encourage prepubertal aggressive chemotherapy/radiotherapy-treated patients without the capacity to generate sperm to cryopreserve testicular tissue. Our previous study has further encouraged us and other to continue this kind of recommendation for prepubertal patients and their families.

## 4. Materials and Methods

### 4.1. Animals

This study was performed in accordance with the Guiding Principles for the Care and Use of Research Animals Promulgated by the Society for the Study of Reproduction. It was confirmed by the Ben-Gurion University Ethics Committee for Animal Use in Research (IL-17-11-2014). Sexually immature seven-day-old ICR (CD-1) (Institute of Cancer Research) male mice (Envigo Laboratories, Jerusalem, Israel) were used.

### 4.2. Busulfan Treatment

Seven-day-old immature male mice were intraperitoneally (i. p) injected with a single dose of busulfan (45 mg/kg; 100 µL/mouse) (BU), which was dissolved with DMSO (1:1 with distilled water). Control mice were injected (i.p) with DMSO alone (1:1 with distilled water). The mice were sacrificed after 3 days–12 weeks of injection in one-week intervals. The body and testes were weighed. The testes were fixed in Bouins’ solution for histological evaluation and/or frozen in −70 °C for protein examination and/or RNA extraction, or were immediately used for tubular cell isolation (after 10 days of BU injection). 

### 4.3. Preparation of Testicular Homogenates

The tunica albuginea were removed. Then, the testes were stored in a protease inhibitor solution (1:250; Sigma, St. Louis, MN, USA) at −20 °C. Thereafter, the tissues were homogenized (with the tissues in ice). The homogenates were centrifuged (13,000 rpm for 15 min at 4 °C), and the upper phase was collected and filtered through a 0.45 µm filter (Merck KGaA, Darmstadt, Germany) and stored at −80 °C.

### 4.4. Isolation of Tubular/Spermatogonial Cells

The tubular cells were isolated from the testes of the BU-treated mice (10 days after BU injection). The testicular cell suspensions were obtained as described by Abu Elhija et al. [35]. Briefly, the testes were decapsulated and mechanically digested by multiple aspirations through pipette tips and a 50-mL syringe containing 20-mL phosphate-buffered saline (PBS). The tubules, which were settled by gravity, were subjected to enzymatic digestion (collagenase type V and DNAse). The suspension of the single cells was filtered through a sterile cell strainer (70 μM; BD Biosciences) and washed with 5 mL MEM (minimum essential media) (centrifugation at 100× *g* for 5 min). After centrifugation, the media were removed and the pellet of the cells in the bottom of the tube was suspended in 1 mL of fresh StemPro-34. The cells were suspended in StemPro-34 and were counted under phase-contrast microscopy in a Neubauer counting chamber. 

### 4.5. Culture of Isolated Spermatogonial Cells in Methylcellulose Culture System (MCS)

The isolated tubular cells were cultured (2 × 10^5^ cells/well/500 µL) in methylcellulose (R&D systems, Minneapolis, MN, USA) (42%) as a three-dimensional (3D) culture system, and 58% of the media were composed of 33% StemPro-34 medium; 10% KSR (knock-out serum replacement) (Gibco, USA); and different growth factors such as human rEGF (recombinant epidermal growth factor) (20 ng/mL) (Biolegend, San Diego, CA, USA), human rGDNF (glial cell line derived nerve growth factor) (10 ng/mL) (Biolegend), human rLIF (leukemia inhibitory factor) (10 ng/mL) (Biolegend), and human r-bFGF (basic fibroblast growth factor) (10 ng/mL) (Biolegend) [38]. The cells were incubated for four weeks in a CO_2_ incubator at 37 °C. Every 7–10 days, we added 50 µL/well of fresh concentrated (× 10) enriched StemPro-34 medium (containing all the growth factors used in the primary culture) to the cell cultures. We tried to match the physiological timing of the development of the cell growth in the culture as closely as possible to that of the mouse spermatogenesis (around five weeks). At the end of the incubation period in MCS, we added 0.5 mL of PBS to the culture wells that contained 0.5 mL MC, mixed by pipetting, and collected the suspension in 15-mL tubes. The tubes were centrifuged at 1600 RPM for 10 min. We removed most of the volume and collected the remainder of around 100 µL from the bottom of the tubes. This volume, which contained the cells, was smeared on a slide for histological examination and/or collected and kept at −70 °C in a lysis solution to be used for RNA extraction.

### 4.6. Testicular Tissues Immunostaining

This was performed as described previously [5,35,38]. Briefly, the testicular tissues were fixed in Bouin’s solution (Kaltek, Italy) and were paraffin-embedded. Sections of 5 µm were placed on superfrost plus slides (Thermo, Braunschweig, Germany) for histological evaluation and for the immunofluorescence staining of SALL4, VASA, and PCNA. The deparaffinated section slides were treated with xylene and ethanol (BIO-LAB, Jerusalem, Israel) for 20 min. After washing with a phosphate buffer solution (PBS) (Biological Industries, Beit HaEemek, Israel), the antigen retrieval of the sections was performed in heated 36% urea solution (Millpore) at in a warm microwave for 5 min (twice). After washing, the nonspecific adhesion sites in the tissues and cells were blocked using a 5% donkey serum (Biological Industries) for 30 min at room temperature. Following the removal of the blocking buffer, the first antibodies were added, as follows: polyclonal rabbit anti-human VASA (Santa Cruz, CA, USA; 1:1000), polyclonal rabbit anti-mouse SALL4 (Abcam, Cambridge, UK; 1:400), and PCNA (Santa Cruz, CA, USA; 1:200). After overnight incubation at 40 °C, the slides were washed, and the specific secondary antibodies were added compatibly to the first antibodies (donkey anti-rabbit IgG (Cy3), donkey anti-goat IgG (Cy3), goat anti-mouse IgG (Rhodamine red); Jackson Immuno Research (West Grove, PA, USA) for 40 min at room temperature. After washing, the slides were dried and DAPI, which stains the nuclei blue, was added to the tissues, and the cover slides were applied. The negative control was incubated in a blocking buffer instead of the first antibody. The specificity of the staining was also examined in the testicular tissue using the relevant IgG isotype as the negative control. The slides were examined for staining using a fluorescence microscope (Nikon Eclipse 50 I; Tokyo, Japan).

### 4.7. Immunostaining of Testicular Cells

The cell immunostaining was performed according to our previous studies [5,35,38]. Briefly, the isolated cells from the mouse seminiferous tubules or those that were collected from the in-vitro culture (MCS) were fixed in cold methanol for 20 min. The process was performed for the immunostaining of testicular tissue, as mentioned above, after antigen retrieval. Following the removal of the blocking buffer, the first antibodies were added, as follows: polyclonal rabbit anti-mouse SALL4 (Abcam, Cambridge, UK; 1:400), polyclonal rabbit anti-mouse VASA (Santa Cruz; 1:100), polyclonal rabbit anti-mouse CD9 (Santa Cruz, CA, USA; 1:100), polyclonal rabbit anti-mouse BOULE (Santa Cruz; 1:50), polyclonal rabbit anti-mouse CREM-1 (Santa Cruz; 1:50), and polyclonal rabbit anti-mouse ACROSIN (Santa Cruz; 1:200). Following overnight incubation at 4 °C, the slides were washed and treated as described for testicular tissue immunostaining.

### 4.8. Hematoxylin Eosin Staining

The cut tissue on the slides was deparaffinized in a K-clear solution 2× for 4 min, and rehydrated in an ethanol concentration 2× ethanol 100% for 2 min each, then 1× ethanol 70% for 2 min and 1 × ethanol 50% for 2 min, and then in deionized water for 2 min. The slides were incubated in a hematoxylin reagent for 1 min, then rinsed in deionized water three times and rinsed in tap water for 5 min to allow for the stain to develop. Next, the slides were dipped in an eosin solution, then in DDW (double distilled water) until the eosin did not stain the water. This was followed by dehydration by rinsing the slides in ascending ethanol concentrations, as follows: 1 × 50% ethanol for 2 min, 1 × 70% ethanol for 2 min, 2 × 100% ethanol for 2 min each, and then rinsed 2 × in K-clear 2 min each. The slides were dried and coverslips using permount were applied.

The slides were rinsed in DDW 2× for 5 min each. Then, 200 µL of periodic acid reagent was added to fully cover the slides, and they were incubated for 10 min. The slides were washed in tap water for 3 min, and the cells were dipped twice in DDW. Then, 200 µl of Schiff’s reagent was added, followed by incubation for 15 min. The slides were washed in running tap water for 10 min, rinsed in DDW for 5 min, and stained in hematoxylin for 1 min. The color was developed in running tap water for 5 min, and dehydrated by rinsing the slides in ascending ethanol concentrations, as follos: 1 × 50% ethanol for 2 min, 1 × 70% ethanol for 2 min, 2 × 100% ethanol for 2 min each, then rinsed 2 × in K-clear 2 min each. The slides were dried and coverslip were applied using permount.

### 4.9. Flow Cytometry Analysis

The isolated cells were suspended in 2 mL ice-cold PBS containing 2% BSA and 1 mM EDTA, and incubated with 0.5–1μg of anti-mouse CD16/CD32 purified per 100 µL for 10–20 min at 4 °C before staining. Then, the cells were incubated with the live/dead dye for 20 min. The cells were washed once in PBS, and stained with anti-THY-1.2 antibody conjugated to Alexa fluor 488, anti-α-6-INTEGRIN antibody conjugated to PE (Phycoerythrin), anti-c-KIT antibody conjugated to APC (Allophycocyanin), and anti-GCSFR antibody conjugated to PercP/cy5.5 at 4 °C for 45 min. Cells were washed once in PBS, resuspended at a concentration of 1–2 ×10^6^ cells/mL, and analyzed on a BD FACS vantage cell sorter. The cells that did not receive primary antibodies were used to gate the positive staining cell population. Live/dead dye was used to gate out the dead cells.

### 4.10. Gene Expression–PCR amplification

#### 4.10.1. Real-Time Quantitative PCR

Isolated cells: Enzymatically isolated testicular cells and developed cells from in-vitro cultures were mixed with 250 μL of lysis buffer and 2-mercaptoethanol mixture (GenElute Total RNA Miniprep Kit; Sigma, St. Louis, USA). The lysates were frozen at −80 °C for later RNA extraction. The cDNA synthesis was performed according to the qScript cDNA Synthesis Kit (Quantabio, Beverly, MA 01915, USA), using random hexamers, and qPCR was performed using specific primers for each examined spermatogenesis marker: gfr-α (forward:CAGTTTTCGTCTGCTGAGGTTG; reverse: TTCTGCTCAAAGTGGCTCCAT; product size, 141 bp), VASA (forward: AGTATTCATGGTGATCGGGAGCAG; reverse: GCAACAAGAACTGGGCACTTTCCA; product size, 83 bp), CREM-1 (forward:TTCTTTCACGAAGACCCTCA; reverse: TGTTAGGTGGTGTCCCTTCT; product size, 138 bp), BOULE (forward: AACCCAACAAGTGGCCCAAGATAC; reverse: CTTTGGACACTCCAGCTCTGTCAT; product size, 163 bp), protamine (forward: TCCATCAAAACTCCTGCGTGA; reverse: AGGTGGCATTGTTCCTTAGCA; product size, 114 bp), ACROSIN (forward: TGTCCGTGGTTGCCAAGGATAACA; reverse: AATCCGGGTACCTGCTTGTGAGTT; product size, 149 bp), and the calibrator gene GAPDH (forward: ACCACAGTCCATGCCATCAC; reverse: CACCACCCTGTTGCTGTAGCC; product size, 450 bp). The qPCR reaction was performed following the 2 × qPCRBIO SyGreen Blue Mix Hi-ROX (PCR Biosystems Ltd., Aztec House, 397–405 Archway Road, London, UK) protocol and was performed using the LightCycler 96 real-time PCR machine (Roche, Roche Diagnostics Corporation, Roche CustomBiotech, Indianapolis, IN, USA). According to the manufacturer introductions for the SYBR Green dye, we ran a PCR profile including a three-step amplification program and subsequent melting, described as follows: preincubation 10 min at 95 °C, 40 cycles of 15 s at 95 °C, 15 s at 60 °C (all the primers were designed with this specific annealing temperature), and 10 s at 72 °C. Melting cycle: 10 s at 95 °C, 60 s at 65 °C, and 1 s at 97 °C. The PCR products were identified and distinguished using the generated melting curve. The “threshold cycle” (*Ct*) values, representing the cycle number at which the sample fluorescence rises statistically above the background and crossing points (CP) for each transcript were defined. The relative quantity of the gene expression was analyzed using the 2^−ΔΔ*Ct*^ method. The results were expressed as the fold of increase related to the GAPDH of the same examined sample.

The microscopy samples were observed with an Olympus IX70 microscope (Olympus, Novato, CA, USA). The digital images and signal intensity charts were prepared using Image-Pro Plus (Media Cybernetics, Bethesda, MD, USA), Microsoft Excel, and Adobe Photoshop 7.0 software.

#### 4.10.2. Data Analysis and Statistical Evaluation

Each culture condition was tested in 4–6 wells. The plotted data are means calculated from 3–10 repeats of the experiment. The standard deviation represents the variability between the independent experiments.
